# Health economic evaluation of digital nursing technologies: a review of methodological recommendations

**DOI:** 10.1186/s13561-022-00378-8

**Published:** 2022-07-06

**Authors:** Kai Huter, Tobias Krick, Heinz Rothgang

**Affiliations:** 1 SOCIUM Research Center on Inequality and Social Policy, University of Bremen, Mary-Somerville-Straße 3, 28359 Bremen, Germany; 2 High-profile Area of Health Sciences, University of Bremen, Bremen, Germany

**Keywords:** Evaluation, Nursing, Technology, Economic, Digital

## Abstract

**Background:**

Health economic evaluation of digital nursing technologies (DNT) is important to provide information that helps avoid undesirable developments and implementations as well as increase the chances of success of developed applications. At the same time, studies and evidence on cost-effectiveness are still very rare in this field. Review studies in related technology areas such as telemedicine frequently criticise the quality and comparability of health economic evaluations conducted in this field. Based on a content analysis of methodological literature on the economic evaluation of innovative (digital) technologies in health and nursing, this article aims to identify specific challenges in this research area and offers recommendations on how to address these challenges to promote more sound health economic evaluations in the future.

**Methods:**

A rapid review was conducted, consisting of a systematic search in the Pubmed database as well as Google Scholar. In addition, the literature lists of the analysed texts were scoured for additional texts to be included. Methodological literature, single studies, and reviews were included. A total of 536 studies were screened, of which 29 were included in the full text analysis.

**Results:**

Based on the systematic content analysis of the studies under consideration, 10 specific methodological challenges are identified, and the methodological recommendations were examined for consideration. A particular focus was given to whether specific methodological approaches might be needed in the context of evaluating the efficiency of DNT.

**Conclusion:**

Many of the challenges identified for the health economic evaluations of digital nursing technologies are comparable to those of other complex health care interventions. The recommendations discussed can help to alleviate those challenges. Future research should focus on alternative approaches to assessing causality in different phases of technology development while maintaining high evidence standards. High-evidence economic assessment of technologies in nursing care should be carried out in routine use, especially if they are intended to be reimbursed by the social insurance.

**Supplementary Information:**

The online version contains supplementary material available at 10.1186/s13561-022-00378-8.

## Background

Digital innovations are expected to change the way both health care and nursing care are provided in the future. Technologies such as robotics, assistive devices, monitoring technologies or decision support systems have already been investigated in nursing care studies [1]. To describe this field of research and the associated technologies Krick et al. introduced the phrase “digital nursing technologies” (DNT) [2], which is also used as a frame of reference for this article. Although a wide range of DNT have been developed and tested in nursing care in recent years, valid evidence on the effectiveness of digital technologies in nursing practice is still scarce [3]. Evidence on cost-effectiveness or efficiency is also rare [1]. Review studies in related technology areas such as telemedicine – a field that has been under research for 30 years now – continuously criticise the poor methodological quality and comparability of health economic evaluations or economic analyses that have been conducted in this field [4–8].

Research on digital technologies in nursing care is funded on the basis of the expectation that their use may increase independency of otherwise care-dependent people, improve the quality of care, increase efficiency, and/or reduce the burden or workload of formal and informal caregivers [9, 10] However, this cannot be taken for granted. Decision-makers who decide on funding or the implementation of digital technologies in health or nursing care need reliable information on which to base their decisions.

Given that there are only very few economic evaluation studies in this field so far, and that research in related fields indicates that the economic evaluation of innovative technologies or new ways of providing health or nursing care may be challenging, this review aims to elaborate on what these particular challenges are – and what approaches or options there are to address them.

Well-established methods of health economic evaluation have been developed in the field of clinical interventions. This raises the question how well these methods are suited for evaluating technological innovations in nursing care – or if they can be adapted for this purpose. We could not identify any prior research on methodological challenges to the economic evaluation of DNT, but it can be assumed that challenges in closely related research fields are similarly applicable here. For this reason, we conducted a review on methodological guidelines for the economic evaluations of digital nursing technologies or closely related areas such as telemedicine, telecare, eHealth, digital health, and mobile health (mHealth) to identify prior research and relevant methodological recommendations.

Terms used to describe or categorise specific technological applications in this area – like eHealth, digital health or mHealth – are often not very clearly defined, they are used incoherently, or categories overlap. Still, the main focus is the application of digital technologies to support health care, nursing care or care-dependent people. Our analysis will focus on methodological challenges that are similar for different fields of application rather than provide distinctive definitions for different areas of technology supported care. We assume that whether certain challenges apply will depend more on the specific application and setting than on a particular category of technology.

The aim of this review is to identify specific challenges of economic evaluations that apply in the field of digital nursing technologies [1, 2] and the recommendations that were developed to date to address these issues.

This article is thus guided by the following research questions:

- RQ1: What specific challenges to the economic evaluation of innovative (digital) technologies in health and nursing are identified in the included studies?
- RQ2: How can these challenges be addressed, and what recommendations are provided in the studies?

### Economic evaluation – basic concepts

Following the common definition by Drummond et al. economic evaluation is defined as the comparative analysis of alternative courses of action in terms of both costs and consequences. Thus, the core tasks of an economic evaluation are the identification, measurement, valuation and comparison of the costs and consequences of the alternatives under consideration [11]. When comparing two alternative health care options the concept of opportunity costs is applied. Opportunity costs are the benefits foregone when opting for one specific intervention over another [12]. This is an important concept to understand because many publications on costs of digital interventions are merely cost analyses and not full economic evaluations [1]. While a simple cost analysis usually focuses only on the financial costs of a given intervention, a full economic evaluation measures the value of an intervention by the value of benefits that were not achieved because resources were not spent on another option. This may include costs whose financial value cannot be determined directly.

As a basis for this article, the most common methods of economic evaluation used in health care are briefly summarised in the following. These methods are differentiated according to how they measure the benefits of an intervention. Cost-effectiveness analysis (CEA) uses single natural parameters to indicate effects of a health intervention, such as for example weight loss, or number of strokes prevented. Cost-utility analysis (CUA) aggregates different aspects in a virtual parameter. The most commonly used parameter is the quality adjusted life year (QALY) that combines a quantitative effect (life extension) and qualitative effects (health-related quality of life) of an intervention. Another option to aggregate different effects of an intervention is a monetary valuation of effects as done by cost-benefit analyses (CBA). CBA values effects based on the assessment of an individual’s willingness to pay (WTP) for it. This allows an individual’s preferences to be captured as part of the analysis, which is in line with the welfarist economic paradigm. Results, however, are strongly dependent on whose willingness to pay is assessed. There is the option to avoid subjective elements by just determining the health and/or nursing care cost avoided – but this would imply that the health gain or gain in quality of life itself would be attributed no value. The presentation of a range of different effects of an intervention without aggregating them in a single measure is called-cost consequence analysis (CCA). This allows us to draw a more comprehensive picture of the effects, but it is left to the decision maker to decide on the relative importance of the different aspects. If it can be reliably assumed that the effects of the two compared interventions are the same, cost-minimisation analyses (CMA) are another option [11].

A brief overview of the main concepts of economic evaluation and their scope of analysis is presented in Table 1.

**Table 1:** Basic concepts of economic evaluation differentiated by outcome measurement

Concept of analysis	Identification/Measurement of Effects	Scope of the analysis
CMA	Effects are regarded as equivalent	Allows only comparison of interventions whose results are considered equivalent
CEA	Single outcome measured in natural parameters	Allows only the comparison of interventions that target the same natural parameter, inadequate to capture multiple impacts
CUA	Aggregated parameter/utility measure, mostly QALY	Allows the comparison of interventions that target different e.g. health effects, but inadequate for capturing impacts beyond health (or the scope of the specific utility measure)
CCA	Multiple endpoints Disaggregated analysis of costs and different effects	Does not provide unique efficiency ratios, allows and requires decision makers to make their own trade-offs between different effects
CBA	Effects are expressed in monetary units, either a) without subjective elements (by avoided health costs) or b) analysis via monetary valuation e.g. of individual willingness-to-pay for it	a) Allows the comparison of different interventions, but disregard effects that are not covered by monetary measures b) Allows the comparison of different interventions and a wide range of effects by capturing individual preferences, but techniques to capture preferences are dependent on who is being questioned and the monetary valuation of health gains is controversial

Own presentation based on Drummond et al. 2005 [11].

Applying these standard methods to the economic evaluation of DNT may be challenging in several ways. To determine more precisely what those challenges are, this study was conducted.

## Methods

### Search process

To identify prior research that discusses methodological aspects of the economic evaluation of digital technologies (including information and communication technologies) applied in health or nursing care we performed a rapid review that consisted of a systematic literature search in PubMed and an extensive search in Google Scholar. In addition, the reference lists of the included texts were scoured for additional studies.

### Eligibility criteria for systematic search

Scientific papers were included that provided guidance and/or discussed in detail methodological challenges or specific methodological aspects of the economic evaluation of the application of digital technologies in health or nursing care – or closely related fields. Papers in English or German language were included, there was no restriction on the publication period. The PubMed-Search was performed on March 8th 2021, the Google Scholar Search was performed on March 9th 2021.

Exclusion criteria:

1. Studies that only commented on single methodological issues in the context of the discussion of study results.
2. Reviews or studies that did not make recommendations or provide explicit guidance on methodological issues related to the application of digital technologies in health or nursing care.
3. Macro-economic concepts or concepts that aim at a rather rough or generalised estimation of the efficiency of technologies.
4. Studies that only provide an exemplary evaluation as a framework for future evaluations.
5. Single clinical trials or RCTs.
6. Modelling studies with no clear reference to empirical data/studies.

These exclusion criteria were chosen to identify papers that systematically address problems in the economic evaluation of particular technologies based on empirical data. Most problems arise from the empirical foundation of the economic analysis, so studies based on rough estimations were excluded.

### Search terms

The search consisted of a fairly specific search in PubMed to keep the number of hits manageable and a more general search in Google Scholar aimed at finding relevant studies not covered by the PubMed search. The search terms can be found in Table 2.

**Table 2:** PubMed Search Strategy

A	“Telemedicine/economics”[Mesh] OR “Mobile Applications/economics” [Mesh] OR “Digital Technology” [Mesh] OR “Independent Living/economics” [Mesh] OR “Ambient Intelligence” [Mesh] OR “Information Technology/economics” [Mesh] OR health-it[Title/Abstract] OR gerontechnology[Title/Abstract] OR ehealth[Title] OR e-health[Title] OR digital health[Title] OR telecare[Title] OR mobile health[Title] OR medical device[Title]
B	((Digital[Title/Abstract]) OR (Technolog*[Title/Abstract])) AND Nurs*[Title/Abstract]
C	(“Cost-Benefit Analysis”[Mesh] OR “Efficiency” [Mesh] OR cost*[Title] OR economic*[Title] OR finance*[Title] OR “Costs and Cost Analysis” [MeSH] OR early assessment[Title/Abstract])
D	(“Research Design” [Mesh] OR “Research/economics” [Mesh] OR “Models, Theoretical” [Mesh] OR guideline*[Title/Abstract] OR framework*[Title/Abstract] OR recommendation *[Title/Abstract] OR method*[Title])

The search was defined as “(#A OR #B) AND #C AND #D NOT clinical trial NOT RCT”.

### Google scholar search

The Google Scholar Search was inspired by Böhler [13] and consisted of 14 different variations of technology-related search terms and economic evaluation-related search terms. Examples are:

- “(Ehealth or mhealth or telemedicine or telehealth or information technology) AND economic evaluation)”
- “Digital-health AND (cost-effectiveness OR cost-utility OR cost-benefit)”
- “(technology AND nursing) AND economic evaluation AND (guideline or framework or methodological)”

For each search, the first 50 hits (sorted by relevance) were screened for relevant publications. As most of the later searches generated no further hits besides duplicates, the search was considered saturated after the 14 variations. A documentation of the search process is provided in Additional file 1.

### Search results

**Fig. 1:**
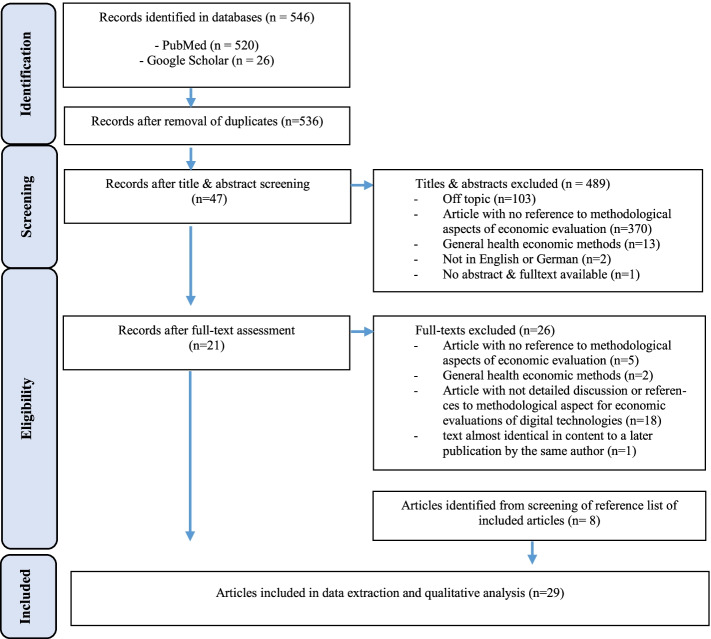
Search results and publication selection process

The PubMed search resulted in 520 studies. The Google Scholar search added 26 studies. After removing duplicates 536 studies remained for title/abstract screening, which resulted in 47 papers whose full texts were screened. Based on the screening of the full texts, 21 studies were included for the final analysis. The screening of the reference lists of the studies included produced a further 8 studies, resulting in a final total of 29 studies included in the analysis. The search and screening process was performed by one author, when in doubt exclusions were discussed and agreed upon with a second author. All results were screened and processed on the basis of the described inclusion and exclusion criteria. The PRISMA flow chart of the search process is provided in Fig. 1.

### Data extraction

Data from the included studies was extracted according to the following categories: author, year, country (first author), technology field, main hypotheses/statements, specific challenges, recommended methods, recommended perspective, recommendations on clinical effectiveness data, multidimensionality, timing, included costs, specific aspects concerning costs, specific aspects concerning outcomes, recommendations on modelling, transferability, additional decision criteria, main future research questions. Based on the extracted information, main contents were summarised in a qualitative content analysis.

## Results

Twenty-nine articles providing guidelines, frameworks, or recommendations on the economic evaluation of health care-related digital technologies, or discussing their specific methodological challenges were included in this review. More than half of them refer explicitly to telemedicine or telehealth (16 studies), three articles refer to eHealth and digital health technologies in general, individual studies refer to mHealth, health information technology, digital health apps, assisted living technologies.

Research reflecting on the health economic evaluation of telemedicine started to appear in the mid-1990s. Up until 2014 most studies in this field focussed on telemedicine applications. Studies on a wider spectrum of digital technologies started to appear after 2015, and 13 of the included studies were published between 2015 and 2020. They cover a wider range of digital technologies and reflect the emergence and broader research on digital technologies in health care in recent years. Table 3 depicts the years of publication and the technology fields of the included papers.

**Table 3:** Years of publication and technology fields

Year of publication	Studies	Technology fields
1992-1998	6	5 telemedicine, 1 digital radiology systems (1992)
2000-2009	7	6 telemedicine, 1 assistive technology
2010-2014	3	2 telemedicine, 1 telemental health
2015-2017	9	telemedicine, telehealth, eHealth, health information systems, health information technology, digital health, assisted living technologies
2018-2020	4	digital health (focus: mHealth, telemedicine), digital health technologies in general, eHealth

The first authors of most papers come from only a few countries: nine from the US, and seven from the United Kingdom. Eight studies were authored by researchers from other European countries (Austria, Finland, France, Italy, Norway, Poland), three papers by Australian, two by Canadian researchers.

Overall, a wide range of challenges is addressed in the included papers*.* The main challenges –addressed by many authors – relate to the rapidly evolving technologies [4, 13–20] and a limited generalisability (external validity) of results [4, 16, 20–23], which is due to a large heterogeneity in the field [19, 23, 24] on the one hand, and a strong context dependency of the interventions on the other [14, 17–19, 24–26].

Unlike most medical or pharmaceutical applications, digital technologies in health and nursing care not only target health objectives, but also imply organisational changes. Alternatively, they may even target mainly organisational processes, but will have effects on health outcomes as well. In this sense, many digital nursing technologies can be categorised as complex interventions often embedded in complex systems (as e.g. hospitals) [13, 27].

We have identified ten challenges (RQ1) and respective recommendations (RQ2) relating to the following key aspects of the economic evaluation that will be described in more detail below.

1. Challenges in performing effectiveness studies
2. Timing of the economic evaluation (iterative approaches)
3. Choice of comparator
4. Choice of perspective
5. Cost Assessment
6. Outcome Assessment
7. Choice of method
8. Transferability
9. Poor quality of economic evaluations – missing guidelines
10. Additional decision criteria

A more detailed description of these aspects, as well as the recommendations discussed in the included articles, is provided below. An extensive overview of the different perspectives is given in Additional file 2.

### 1. Challenges in performing effectiveness studies

A key precondition for assessing the cost-effectiveness of an intervention is the availability of valid data on the effects or effectiveness of an intervention. Randomised controlled trials (RCTs) are usually regarded as gold standard to provide effectiveness data – this is corroborated by many of the included authors [13, 14, 21, 26, 28–30]. The lack of RCTs or the general lack of reliable evidence on effectiveness is considered a challenge by several of them [5, 21, 31]; often small or inadequate sample sizes limit the validity [4, 15, 21]. In many areas, the development and introduction of a technology may be incremental, i.e. the application or system may change regularly due to updates, extensions of functionalities, etc. [26]. As RCTs are difficult to perform in this context, a significant number of authors question this standard [16, 17, 19, 24, 26, 27, 32]. While on the one hand time frames of RCTs are often considered too long in the light of the rapid or continuous development of technologies, on the other hand usage patterns – and thus effectiveness – may be different from those under study conditions. Bergmo calls for pragmatic or naturalistic trials as gold standard for telemedicine studies [19, 24], McNamee et al. recommend natural experiments or Cluster-RCTs as a database for studies on digital health interventions [27], while others demand more flexible or additional approaches [17, 26, 33].

### 2. Timing of the economic evaluation – iterative approaches

The challenging task of performing RCTs – or other types of effectiveness studies on digital heath technologies – raises the question of the right timing for economic evaluations.

As reliable RCTs and especially long-term evaluation studies are often scarce [21], it has to be assumed that the short-term effects of evolving technologies differ significantly from long-term effects [15]. Several authors recommend an *iterative approach*: Ohinmaa et al. recommend the performance of a series of rapid, less detailed evaluations to provide decision makers with timely interim advice [16]. Luzi et al. and Sisk & Sanders refer to the necessity of an ongoing continuous assessment [26, 34]. McIntosh and Cairns recommend that the economic evaluation be incorporated into the clinical study at the beginning of the trial, relevant costs collected, and a sensitivity analysis then be carried out with updated costs at the end of the study [15]. LeFevre et al. present a stage-based model that advises the use of different economic methods depending on the maturity of the technology. (Pre-)prototype phases should be accompanied by model-based economic evaluations. During pilot and effectiveness studies economic evaluations should be based on primary data. In order to predict long-term effects modelling techniques can, again, be used [32]. This approach is also recommended by Böhler [13].

### 3. Choice of comparator

The choice of comparator may be challenging [25, 35]. If no obvious alternative course of action exists, the usual comparator will be ‘standard care’, but data on conventional (administrative) services may be difficult to assess [18]. For some technological innovations the alternative may only be inaction. Especially from a social perspective the social costs of inaction may be difficult to determine, as for example the social cost of no health care in a region where – without telemedicine or telecare – medical care would not be available at all, or a care dependent person would have no other option for support [22, 36].

Another recommendation refers to the adaptability/customisability of some technological interventions: if the implementation of the technological intervention may be adapted to specific situations, e.g., with different configurations of the technology, different options would have to be included in the analysis [4, 13, 26, 37].

### 4. Choice of perspective

The choice of perspective is decisive for determining which costs and effects are considered in an economic evaluation. The decision on the perspective may be problematic, as there may be a range of different stakeholders involved in the implementation of an innovative digital intervention and the recommendations in the included studies differ considerably. While a number of them merely point out that the perspective has to be stated clearly [14, 24, 37] or depends on the research question [18, 26, 32] or decision-maker [17, 28], some authors – mainly in recommendations on the economic evaluation of telemedicine – clearly recommend the application of a societal perspective [23, 34, 38]. Another range of studies recommends considering the fact that there may be different parties involved and advocates. These recommend a multistakeholder perspective [4, 13, 16, 22, 30], or complementing a societal perspective with an analysis of costs and benefits that is differentiated according to different stakeholder groups [15, 21].

### 5. Cost assessment

There is a range of challenges pertaining to the assessment of costs. The main challenges specific to digital technologies are outlined/listed below^1^:

- ▪ Continuing changes in the price-performance ratio of equipment and related software due to rapidly changing prices of technology, making costs difficult to estimate [15, 19, 23, 39].
- ▪ Costs may be dependent on uptake or usage rates, which are difficult to foresee. Thus, due to existing fixed costs, costs per unit of service would be high for low caseloads but decline with increasing volume of use [5, 15, 22, 26, 40].
- ▪ Lacking evidence on costs, especially on costs that are difficult to measure [4, 5, 16, 18, 26].
- ▪ There may be a variety of budgets of different stakeholders affected, and these should all be considered [4, 13, 21], especially as costs and cost-savings may fall into different budgets [22].
- ▪ Divergent opinions on the classification of costs [26, 39] and the inclusion of research and development costs [20].
- ▪ Some of the equipment may be used for other applications as well, thus boundaries of the intervention may be ambiguous, or due to multiple use of the technology it can be difficult to determine whether costs should be attributed to an intervention or not, e.g. the implementation of a wireless network [13, 14, 17, 23, 34].
- ▪ Depreciation periods may be short, and life cycles of technologies have to be considered as well as costs for regular updates [20, 22, 27].
- ▪ There may be large differences between costs for pilot projects and the costs of mature real-life applications; it therefore makes more sense for costs to be assessed in pragmatic trials [13, 24, 39].
- ▪ Some authors highlight that the costs of supporting health care providers in the use of eHealth interventions, e.g. costs of training, helpdesks, change management [4, 19] and the costs of assessing what type of technical support the client needs [36] should be included as well. As the implementation of technology may change, organisational processes or changes in clinical pathways may also give rise to indirect or intangible costs (e.g. changes in staff morale, new types of staff) that have to be considered [25, 30].

Recommendations for addressing these challenges are that costs be reported in very transparently; quantities of resources and cost weights be reported separately to facilitate transferability to other contexts [13, 19]. In addition, several authors recommend an iterative approach that regularly updates the cost-assessment throughout the different stages of a technology’s life cycle [13, 16, 26, 34, 40]- and the use of micro-costing methods if no pricing information is available [20]. A more general recommendation is the performance of sensitivity analyses for the (many) remaining areas of uncertainty, e.g. anticipated changes in equipment and transmission costs [5, 14, 15, 23, 34].

### 6. Outcome assessment

One main challenge pertaining to the outcome assessment are the already mentioned difficulties in assessing effectiveness data, respectively the lack of (good-quality) evidence on effectiveness outcomes [4, 16, 18, 23, 30, 40]. Another challenge mentioned by many authors relates to the fact that digital health or nursing interventions may have diverse or multidimensional impacts, while the most widely used health economic evaluation methods – especially CEA and CUA – are rather inadequate for capturing multiple impacts or impacts beyond health [4, 17, 19, 25, 28, 33, 35].

This entails various difficulties. Digital technological interventions in health and nursing care not only address health outcomes, but also imply or explicitly address organisational change processes or individual behavioural change processes that may not lead to immediate health effects. Kolasa & Kozinski – to cite one example – suggest differentiating clinical, organizational, behavioural and technical impacts [25]; Bongiovanni-Delarozière et al. recommend the assessment of four categories of outcomes indicators for telemedicine interventions, namely accessibility, professional practice/care organisation, care quality, and costs [4]. On the one hand this may compound the difficulty of measurement and valuation of non-health outcomes [13, 19, 28, 35, 38]: standardised measures are often missing [14], some outcomes may be intangible or difficult to quantify [17, 22, 30, 33], or sometimes only intermediate (surrogate) outcomes may be accessible, while the relationship to health-related measures is not well-established [16, 23, 27].

On the other hand, these effects may accrue to different stakeholders [19, 21, 30], which may raise the question how to weight outcomes for different stakeholders, or how to decide between divergent preferences of different stakeholder groups [35].

Overall, 17 of the included texts explicitly indicate the need for a multidimensional outcome assessment, but recommendations on how to do this differ. Two studies recommend the use of CBA [21, 38] to deal with these challenges, another one the combination of CUA and CBA [35]. This implies a monetary valuation of different outcomes to enable the comparison of diverse or aggregated outcomes. This is rejected by other authors, who consider a monetary valuation of health outcomes or QALYs as problematic or even ethically inappropriate [13, 15, 19, 36]. Luxton concludes that there is a need for standardised effectiveness outcomes for telemedicine that include clinical outcomes as well as other factors such as patient compliance and treatment satisfaction [14]; the British National Institute for Health Care Excellence (NICE) advises the use of CCA if applicable [41], as do McIntosh & Cairns, who suggest the use of a balance sheet with disaggregated costs and benefits and the use of conjoint analysis for the valuation of non-health benefits [15].

Four authors recommend the use of an outcome matrix differentiated by stakeholders and different outcome categories to assess the multidimensional outcomes [4, 16, 30, 42]. Böhler advises the use of CEA and CUA as a reference case – and the consideration of additional methods such as multi-criteria decision analysis (MCDA) or choice-based methods if there is a significant non-medical benefit. Similarly, Kolasa & Kozinski propose a weighting of the different value attributes based on the preferences of chosen stakeholder groups [25].

### 7. Choice of method

Regarding the general choice of method and the applicability of the established methods of health economic evaluation, many authors recommend the use of the established methods (CEA, CUA, CBA, sometimes CCA) without any further adjustments, mainly indicating that the choice of method depends on the research question [5, 14, 19, 23, 26, 28, 34, 37, 41]. Another large group focusses rather on the need for an extension of the established methods to cover multidimensional outcomes that accrue to different stakeholders [4, 13, 16, 18, 25, 30, 40]. Explicit use of CBA to cover multiple outcomes is recommended by very few authors [21, 35, 38].

Additional methods that are recommended mainly by individual authors are:

- ▪ Extended CEA and Net benefit regression CEA for subgroup analyses [32],
- ▪ Social audit analysis [16, 30, 40],
- ▪ Multi-criteria decision analysis [13, 25],
- ▪ Economic production functions, decision making frameworks, functional economic analysis [17],
- ▪ Decision theoretic approaches, social network analysis [27],
- ▪ Q-methodology to elicit group views on relevant attributes [35],
- ▪ Conjoint analysis for the valuation of non-health benefits [15],
- ▪ Budget impact analysis [28, 38, 41],
- ▪ SCAI – an instrument for cost assessment that is presented in the pertinent article [43],
- ▪ Multivariate statistical techniques to improve efficiency of estimation and adjustment of selection bias [42].

### 8. Transferability of results

A major challenge is the question of the external validity or transferability of the results of the evaluations performed. A strong context dependency or sensitivity of the results of economic studies is stated in several of the included texts [14, 17–19, 24, 25]. This may be caused, for example, by differences in the specific technologies or equipment used [19, 23, 24], local adjustments in the implementation of specific technologies [26], or variations in willingness to use by end users [25]. As many technological applications may alter organisational routines or working patterns, the effects may differ greatly depending on previous work patterns and organisational structures [22, 24], or organisational competence [24]. Structural aspects of the national health care system or regulatory changes may also influence the use and possible effects [25–27].

Recommendations for dealing with these questions are:

- ▪ a thorough reflection on generalisability, in case of minor differences between settings and interventions a transferability of results is possible [19],
- ▪ a reflection on the transferability of study results to different settings early on in the design of the evaluation study; this includes theoretical considerations on the relevance of potential variability factors and their exploration e.g. by subgroup, sensitivity and scenario analyses [13],
- ▪ a replication of studies in different countries to assess differences in organisational and funding aspects [30],
- ▪ discussion of results of economic evaluations, focusing on their generalisability [4],
- ▪ evaluations based on pragmatic trials, estimation of different variations of relevant context variables by decision modelling [24].

### 9. Poor quality of economic evaluations - missing guidelines

Many of the included texts criticise that there are only few economic evaluations on telemedicine or other digital innovations in health care, that the quality of published evaluations is rather poor, and that existing guidelines and methodological recommendations tend to be disregarded [4, 5, 14, 17, 18, 21, 32, 35]. Thus, conducting studies – and especially high-quality studies that follow the general established guidelines for health economic evaluations – seems to be a challenge in itself. On top of that, several authors point to the drawback– or reason for the poor quality – that guidelines for this specific field of research have been lacking so far [14, 20, 25, 32]. In fact, several of the included texts try to fill this gap and develop frameworks or present guidance tutorials for future evaluations.

In order to improve this situation, many recommendations revolve around guidelines – that general guideline compliance should be increased and the specific recommendations presented in the different publications should be followed. Some authors indicate a further need for the development of common guidelines or frameworks [13, 14, 20, 25, 27] or the development of additional standard parameters for the valuation of certain costs or benefits [35, 36].

Reardon, specifically, points out that research on necessary consensus guidelines is unlikely to be financed by individual research or provider groups [17], which indicates the need for adequate funding of corresponding research.

### 10. Additional decision criteria

Some of the texts highlight additional decision criteria to be taken into account, in particular equity and/or distributional consequences [15, 28, 30, 32, 35, 40], accessibility [4, 15], ethical and legal aspects [4], priorities/values of policy-makers [16], or “clinical experience, common sense and professional ethics” [36].

## Discussion

This review reveals a wide range of challenges to the economic evaluation of digital interventions in health care that are also applicable to digital nursing technologies (RQ1). Reflection on some of these problems date back to 1992. There has been a huge debate since the 1990s focussing on telemedicine – and many of the challenges identified at an early stage persist today. The related field of telenursing has become particularly relevant in recent years as the COVID crisis has necessitated new methods of remote care [44]. This makes challenges and recommendations on the health economic evaluation of telemedicine applications equally relevant for the field of nursing. Most of the findings from the included articles are also very generic and can therefore be reflected in the context of nursing.

At the same time, recommendations on how to tackle or approach these challenges (RQ2) differ widely as well. While some authors conclude that conventional techniques of economic evaluation are inappropriate for assessing e.g. telemedicine applications [15], or only partially applicable [4] – other authors rather detect a problem of missing guidance on which analytical approaches are most appropriate [14, 32], or recommend the further development of the methodology [25, 35].

Several of the included texts present or develop guidance frameworks and tutorials for future evaluations. Some of these mainly present general guidelines or recommendations on health economic evaluation and do not or hardly account for specific challenges to digital technological interventions [5, 26, 34, 37]. But there are several frameworks or recommendations that go beyond this and provide guidance on the specific, reported challenges of digital interventions [4, 13, 15, 21, 28, 32, 41].

In short: the included papers, the authors’ opinions on whether and which health economic methods can and should be used – or whether they should be developed further – are highly diverse.

A total of 10 challenges were identified, four of which can be described as the major ones in the context of digital nursing technologies. These are:

1. the strong context dependency of the interventions
2. the multidimensional effects on different stakeholders
3. the incomplete assessment of economic costs, different approaches on the measurement and valuation of cost
4. the rapid and often incremental development of the technologies, that may result in the need to repeatedly adapt evaluation results.

The economic evaluation of technological interventions in health and nursing care shares many methodological problems that are discussed in the context of the economic evaluation of complex interventions or public health interventions that are similar in this respect [45–49]. This refers especially to the strong context dependency of the interventions and the possibility of multidimensional outcomes beyond health. In the field of DNT there are specific outcomes for different nursing-relevant stakeholder groups, e.g. professional caregivers as well as informal caregivers or the persons in need of care themselves [2, 50]. These perspectives and the specific outcomes, as well as specific cost measurements, need to be brought together for understanding, collecting, and using cost estimates in a DNT multi-stakeholder perspective. In particular, conflicts in prioritising specific groups and their benefits must be taken into account [51].

One critical debate about the use of MCDA combined with economic evaluations is very pertinent here [52–56]. Baltussen et al. conclude that MCDA is often used inadequately in economic evaluations because opportunity costs are not considered [53]. For this reason, Marsh et al. recommend complementing MCDA with an economic evaluation, but not substituting the economic evaluation for MCDA [56]. Still, these seem to be interesting approaches to integrating multidimensional outcomes that should be developed further.

There is an extensive methodological debate on the evaluation of complex interventions. Implementing DNTs is always part of a complex intervention as it consists of different individual interacting components such as technology characteristics, the implementation process, the nature and characteristics of the intervention setting, or the characteristics of the individuals involved. These components can act independently of each other or interdependently, which makes the explanation of causality difficult and thus represents a challenge for the DNT evaluation process [57].

The question which components or combination of components lead to an outcome is not easy to answer [46]. The process of implementing the DNT and the systematic understanding of the process is particularly relevant here. The complexity of the technologies and the complexity of the implementation process influences whether or not the introduction process is successful [58]. Nursing homes often don’t strategically use a systematic process for implementing technologies, give their staff inadequate support and training for the technology implementation, and often have a poor infrastructure for fostering implementation [59], making the systematic economic evaluation of DNT difficult.

Staff shortages in nursing homes, putting tremendous pressure on existing staff in countries like the US [60] or Germany [61], can be a further challenge to the implementation of DNT. Although the implementation of technologies such as telenursing should ideally solve or alleviate this problem, the process of technology introduction is still demanding as it requires the involvement of staff for additional organisational and administrative tasks or causes interruptions in the daily nursing routine, thus impacting the nursing process [61]. Staff shortages, high-pressure situations and the extremely demanding task of implementation can be a major barrier to DNT usage and evaluation, especially if knowledge of the DNT is not easily available and its application difficult to learn [62]. Such procedural and staffing challenges described above also pose barriers to the health economic evaluation of DNT. Still, this debate also holds further aspects referring to general aspects for evaluating complex interventions, like the necessity of process evaluations of the way in which an intervention was implemented, to understand why an intervention fails or works [63–65].

Challenges pertaining to the incomplete assessment of economic cost, or different approaches in the measurement and valuation of cost are not specific to the evaluation of DNT, but rather a common problem of economic evaluations in other areas as well [66, 67]. However, criticism of the poor methodological quality of economic evaluations in the field of telemedicine or digital health technologies is very persistent. Conducting economic evaluations according to the existing methodological standards of the discipline – including a comprehensive assessment of all relevant cost categories – seems to be a challenge in itself. The adequate identification of relevant cost categories should be achievable by complying with general economic evaluation guidelines, but the actual assessment may be more challenging. Sufficient funding for economic evaluations is necessary to ensure the conduct of high-quality evaluation studies. Economic evaluations should be planned at an early stage of the study design to ensure that all relevant data are collected. The development of guidelines for specific fields of application of technologies could be helpful to align evaluations and thus obtain more comparable studies.

The most specific challenge to the economic evaluation of digital nursing technologies, which might necessitate adapted procedures, is their rapid and often incremental development. An extreme example are artificial intelligence technologies. These technologies are rapidly evolving and self-learning, which makes it difficult to determine their effectiveness at any given point in time. Especially in the context of regulatory and reimbursement requirements in nursing, this challenge is currently particularly critical because the effectiveness measurement is tied to a certain state of the technology and does not allow for further development.

An iterative approach as proposed by LeFevre et al. [32] and Böhler [13] seems recommendable and might be worthwhile here. The economic methods used should then be adapted to the stage of development of the technology. Such an approach also opens pathways into the discussion of risk-sharing-models: As risk generally decreases over the course of the evaluation, different risk distributions are possible. Risk sharing as traditionally known from the pharmaceutical industry could also be applied to the field of digital nursing technologies if health outcomes are linked to payments [68]. This could ensures that people in need of care and payers get the best technology possible for their money by only paying for what really works [68] and technology manufacturers can enter a marketplace while evaluating value in a real-world setting. “Early stages of development can be accompanied by model-based evaluations, which allow an initial cost effectiveness assessment and the strategic development of a business case. Pilot studies can be used to further explore possible costs and consequences of the technological innovation. Full economic evaluations are complex and costly; they should be based on high-quality effectiveness studies to ensure that valid data on the effects of the intervention is obtained. This would only be worthwhile once the technology has reached a certain level of maturity. Technological changes that affect the prices of the technological component of the interventions may be included in sensitivity analyses, and their retrospective integration should be fairly straightforward. Technological changes that significantly change the performance of a technology, however, should be tested in pilot studies first. If they can be expected to significantly affect the outcomes, these changes may also be assessed by sensitivity analyses. Smaller, less costly study designs are entirely appropriate in early stages of digital technology evaluation [69]. However, the authors of this paper particularly advocate high evidence standards when it comes to adoption into standard care by social insurance providers. Methodological evidence standards should not be lowered simply to facilitate reimbursement. Added value and measurable effectiveness, as well as, e.g., cost-effectiveness should be demonstrable. Appropriate funding should be made available to support studies with high standards of evidence.

### Limitations

The search process and analysis process were carried out very thoroughly, but there is always the possibility that some articles were overlooked. However, we assume that the main challenges and recommendations in the included texts have all been identified and that no problems have arisen as a result. It was also noticeable that none of the included texts described their own limitations, which is not uncommon in methodological texts, but certainly worth pointing out for critical reflection.

## Conclusion

Conducting an economic evaluation in the context of digital nursing technologies is complex, but important. Many of the described challenges are comparable to the challenges in similar technological fields and other complex health care interventions. The recommendations discussed from the literature can help mitigate these challenges and should encourage the timely economic evaluation of digital care technologies.

The information obtained from health economic evaluations is very important and may help to avoid undesirable developments in research and development and increase the chances of success of the applications developed for nursing care.

Performing such economic evaluations can be costly, but the availability of important information at an early stage of development provides many opportunities to initiate appropriate steps towards their further development. The costs associated with missteps at a later stage can be much higher than conducting a sound preliminary economic evaluation at an earlier stage.

Future research should focus on the advancement of alternative approaches to assess causality [17] and keep up with the pace of technological advancement while still maintaining a high quality evidence standard in different phases of development. A rolling evidence procedure over time with a high standard of evidence, as in the German Fast Track Process for Digital Health Applications (DiGA) [70], with simultaneous collection of economic parameters could be a future option for economic evaluations.

As part of a stage based approach economic evaluations of technologies should also be carried out in routine use [36, 40], and not just in laboratory or experimental study settings. It is essential to thoroughly discuss and reflect on the contextual conditions of the results in order to enable assessments on transferability to other settings or countries [30].

.Overall, the information in this article can be used as a basis for methodological discussion and the further development of health economic evaluations in the area of nursing technologies as well as the application of methods best suited to the stage of development of the respected digital tool.

## Supplementary Information

**Additional file 1.** Documentation of the search process

**Additional file 2.** Detailed analysis of the articles

## Data Availability

The datasets used and/or analysed during the current study are available from the corresponding author on reasonable request.

## Abbreviations

BIA: Budget Impact Analysis
CBA: Cost-Benefit Analysis
CCA: Cost-Consequence Analysis
CEA: Cost-Effectiveness Analysis
CMA: Cost-Minimisation Analysis
CUA: Cost-Utility Analysis
IT: Information technology
DiGA: Digital Health Applications
DNT: Digital Nursing Technologies
mhealth: Mobile health
RCT: Randomised Controlled Trial
QALY: Quality-adjusted life year
US: United States
WTP: Willingness-to-pay

## Footnotes

1 Challenges that were mentioned by more than one text.
