# Supplementary material for: Health economic evaluation of digital nursing technologies: a review of methodological recommendations
Source: Health Econ Rev. 2022 Jul 6;12:35. doi: 10.1186/s13561-022-00378-8 (PMC9258051; doi:10.1186/s13561-022-00378-8)
Supplement: Supplementary file 1 — Additional file 1. Documentation of the search process [file 13561_2022_378_MOESM1_ESM.docx]

**Additional File 1: Search Process**

**Pubmed-Search (08-03-2021)**

| **Search** | **Query** | **Results** |
| --- | --- | --- |
| 9 | #8 NOT (Clinical Trial[Publication Type]) | 520 |
| 8 | #6 OR #7 | 617 |
| 7 | #2 AND #5 | 211 |
| 6 | #1 AND #5 | 422 |
| 5 | #3 AND #4 | 64,389 |
| 4 | ("Research Design" [Mesh] OR "Research/economics"[Mesh] OR "Models, Theoretical" [Mesh] OR guideline*[Title/Abstract] OR framework*[Title/Abstract] OR recommendation*[Title/Abstract] OR method*[Title]) | 3,450,533 |
| 3 | ("Cost-Benefit Analysis"[Mesh] OR "Efficiency"[Mesh] OR cost*[Title] OR economic*[Title] OR finance*[Title] OR "Costs and Cost Analysis"[MeSH] OR early assessment[Title/Abstract]) | 359,155 |
| 2 | ((Digital[Title/Abstract]) OR (Technolog*[Title/Abstract])) AND Nurs*[Title/Abstract] | 14,482 |
| 1 | "Telemedicine/economics"[Mesh] OR "Mobile Applications/economics"[Mesh] OR "Digital Technology"[Mesh] OR "Independent Living/economics"[Mesh] OR "Ambient Intelligence"[Mesh] OR "Information Technology/economics"[Mesh] OR health-it[Title/Abstract] OR gerontechnology[Title/Abstract] OR ehealth[Title] OR e-health[Title] OR digital health[Title] OR telecare[Title] OR mobile health[Title] OR medical device[Title] | 14,348 |

**Google Scholar Search (09-03-2021)**

| Search | **Search Terms** | **Number of new hits** | **Dupli-cates** |
| --- | --- | --- | --- |
| 1 | (Ehealth or mhealth or telemedicine or telehealth or information technology) AND economic evaluation) | 11 |  |
| 2 | (Ehealth or mhealth or telemedicine or telehealth) AND (cost-effectiveness OR cost-utility OR cost-benefit) | 0 | 4 |
| 3 | (Ehealth or mhealth or telemedicine or telehealth) AND (decision analysis Or economic OR multicriteria) | 0 | 4 |
| 4 | (Ehealth or mhealth or telemedicine or telehealth) AND (reimbursement OR adoption) | 0 | 3 |
| 5 | ehealth or guideline or evaluation or good practice or economic or provision or reimbursement | 5 | 4 |
| 6 | (technology AND nursing) AND economic evaluation | 0 |  |
| 7 | (technology AND nursing) AND economic evaluation AND (guideline or framework or methodological) | 1 | 1 |
| 8 | Gerontechnology AND economic evaluation | 0 |  |
| 9 | Artificial-Intelligence AND economic evaluation AND (health or nursing) | 0 |  |
| 10 | Digital-health AND economic evaluation | 4 | 1 |
| 11 | Digital-health AND (cost-effectiveness OR cost-utility OR cost-benefit) | 1 | 3 |
| 12 | Telecare AND economic evaluation | 1 | 3 |
| 13 | Assistive-Technology AND economic evaluation | 2 |  |
| 14 | Assistive-Technology AND (cost-effectiveness OR cost-utility OR cost-benefit) | 1 |  |
|  | **Total** | **26** |  |
