# Supplementary material for: Health economic evaluation of digital nursing technologies: a review of methodological recommendations
Source: Health Econ Rev. 2022 Jul 6;12:35. doi: 10.1186/s13561-022-00378-8 (PMC9258051; doi:10.1186/s13561-022-00378-8)
Supplement: Supplementary file 2 — Additional file 2. Detailed analysis of the articles [file 13561_2022_378_MOESM2_ESM.docx]

| **Author/ Year** | **Technology** | **Text type** | **Special challenges** | **General methods applicable** | **Recommended methods** | **RCTs recommended?** | **Complex/multidimensional** | **perspective** | **Specific for costs** | **Additional decision criteria** | **Multidimens. outcome assessment** | **Specific for outcomes** | **Which outcomes** |
| --- | --- | --- | --- | --- | --- | --- | --- | --- | --- | --- | --- | --- | --- |
| Andrich, Caracciolo 2007 | assistive technology | Tutorial | Assessment of social costs averted by individual AT is necessary if no technological alternatives are available | partially | the proposed instrument for cost assessment, which should be used as informative tool, not as decision-making tool; costs and effectiveness outcomes should be analysed separately | n.m. |  | social perspective (social costs) | detailed assessment of social-cost of non-intervention is necessary as long as no technological alternatives are available | clinical experience, common sense and professional ethics | yes | comprehensive outcome indicators for individual AT at the population level are missing so far; many effectiveness outcomes cannot be measured in monetary values (or the translation raises ethical issues) thus CBA should be avoided | possible outcomes for all stakeholders should be identified (individual/family/professionals/community) |
| Bashshur 1995 | telemedicine | Discussion | multiplicity of outcomes in telemedicine; CEA and CBA emphasize measurable costs and benefits, thus considerations of e.g., equity are deemphasized | partially | CEA or CBA; inferential statistics/multivariate statistical techniques to improve efficiency of estimation and adjustment of selection bias | RCTs, complemented by multivariate statistical techniques |  | client, provider and societal perspective | distinction between capital an operational cost may be critical for telemedicine because of different funding sources; i.e., operational costs may be fixed based on maintenance contracts or variable based on actual usage |  | yes | proposes a matrix (client/provider/society - accessibility/costs/quality) to analyse the wide range of possible effects | differentiates between accessibility, cost effects and quality effects (relating to processes of care, outcomes and interpersonal aspects) that may accrue for clients, provider or society |
| Lau 2017 | e-health (HIS) | Framework, Review | not discussed | yes |  | n.m. |  | should be clearly stated (individual, organization, payer, society) |  |  | n.m. |  | financial outcomes (including changes in revenues, labour and supply costs, capital costs expressed in monetary units); non-financial outcomes (including changes in resource utilization and health outcomes in their natural units). |
| Reardon 2005 | telemedicine | Discussion (incl. Review) | low level of use, rapidly changing technology, large impact of contextual factors; ambiguous boundaries of the interventions; broadly defined or intangible outputs; to identify the decision-making information requirements; to depict the decision framework that defines the contextual validity of findings; | partially | economic production functions, decision making frameworks (incl. cost assessment frameworks), analysis of environmental determinants, functional economic analysis (FEA) | partially (not always the best/more flexible methods necessary) | yes | perspective of the decision-maker should be the main focus; other perspectives may be included to provide more generalizable results | tools and schemes of clinical research (evaluations of drugs or devices) are not directly applicable |  | n.m. | outcomes are often difficult to identify, because detecting them depends on prior expectations/knowledge; whether an outcome is a benefit may depend on the perspective of the decision maker or stakeholder; assigning value to outcomes and aggregating different outcomes/benefits is a difficult task. | a detailed analysis is necessary (based on productions functions) to identify possible positive effects (reduction of travel time, improved care, reduced use of medical care etc.) |
| Crowe 1998 | telemedicine | Framework for cost assessment | divergent opinions regarding the classification of costs; continuing changes in the price–performance ratio of equipment and related software; differences in costs for pilot projects vs. mature real life applications | n.m. | n.m. | n.m. |  |  | divergent opinions regarding the classification of costs; continuing changes in the price–performance ratio of equipment and related software; differences in costs for pilot projects vs. mature real-life application |  | only costs considered | only costs considered | only costs considered |
| Kolasa; Kozinski 2020 | digital health (telemedicine, mobile health) | Review, Recommendations | multidimensional impact of DHIs, determination of the appropriate alternative course of action, dependency on many context factors (including willingness to use by end-users, healthcare systems' capacity to benefit from the innovation) | partially | MCDA | n.m. | yes | multistakeholder-perspective | infrastructure investments and disinvestments, effects of changes in clinical pathways have to be considered | multidimensional decision | yes | multiple dimensions have to be considered | clinical, organizational, behavioural and technical impacts |
| Lobley 1997 | telemedicine | Discussion | Effects are difficult to analyse as the introduction of telemedicine might change working patterns and cause structural changes in the medical field, costs are dependent on the uptake which is difficult to foresee and will be dependent on reimbursement schemes. Knowledge gained in "telemedicine experiments" is difficult to transfer to real life settings; some of the benefits are difficult to quantify | not discussed | n.m. | n.m. | yes | multistakeholder-perspective (differentiated by the different parties involved) | depreciation periods are short, unit costs are highly dependent on the number of consultations, large variety of potential savings; cost and cost-savings may fall in different budgets | diverse possible benefits considered | n.m. | Diverse possible benefits, some of the benefits are difficult to quantify. | i.e. improved treatment, faster/more accurate diagnosis, reduced need for patient referral, improved training and education, reduced disruption to patients through reduced travel, reduced need for specialist consultation due to knowledge transfer etc. |
| Luxton 2013 | Tele-mental health | Discussion | complex cost factors and uncertainties that are associated with aspects of healthcare delivery and technology, rapidly evolving technology; high context-sensitivity, missing guidelines and standardized measures for costs and benefits | yes | CEA, CUA, CBA, CMA, uncertainties should be addressed in sensitivity analyses | yes | yes | should be clearly stated | utilization rates of the equipment influence the offset of the costs; equipment may be used by other applications as well |  | yes | lack of agreed-on outcomes and standardized measures for THM; Standardized outcomes are needed that include clinical outcomes as well as other factors such as patient compliance and treatment satisfaction | primary outcome: health related quality of life |
| Luzi; Pecoraro; Tamburis 2016 | health information technology | Tutorial | health IT supports care processes (diagnostic, treatment/therapy, nursing) and auxiliary processes (i.e. appointing making, documentation), causal relations are difficult to determine and measure;  difficult identification of costs; incremental development of many health IT solutions; locally adjusted implementations; measurement of benefits that depend on how the system is used, the organization and medical context and aspects of the national health system; outcomes have to be measured in terms of changes in the health care and management processes (as they not directly affect health states) | yes | CEA, CUA, CBA, CMA, CCA, depending on research questions, viewpoint of decision maker an data availability | partially | yes | depends on research question | some costs are difficult to measure or subjectively attributed to different classes of costs. | multidimensional outcomes | may be necessary | as health IT does not directly affect health states, benefits have to be measured in terms of changes in the health care and management processes | outcomes should be differentiated by distinguishing the main functionalities: 1) capturing, storing, managing; sharing data 2) informing and supporting clinical decision-making 3) delivering expert professional and or consumer care remotely |
| Ohinmaa; Hailey; Roine 200 | telemedicine | Review | difficulties in performing meaningful effectiveness studies; challenges in assessing cost and effectiveness data; assessment is difficult due to changes in technology and healthcare systems; lack of resources for funding evaluation studies, limited generalizability of results | partially | performance of a series of rapid, less detailed evaluations to provide decision makers with timely interim advice; analysis of results by "social audit analysis" | partially | yes | multistakeholder-perspective | n.m. | multidimensional outcomes, priorities/values of policy makers | yes | often only surrogate outcomes available, investments in telemedicine will be accompanied by changes in patterns of care - some of them may be very specific, so generalizability is limited | Diagnostic quality, health related QoL, Clinical changes in health, changes in management process in hospital, increasing know-how in PC, non-health outcomes of a patient (certainty, access of care, equality) |
| Snoswell; Smith; Scuffham; Whitty 2017 | telehealth | Discussion | covering extra-clinical benefits of telehealth interventions (non-health and process-related outcomes) | yes | CBA, BIA | n.m. | yes | societal | n.m. | are covered by CBA | by CBA | next to validated questionnaire tools informal methods such as free-form questions should be included to capture specific additional data | societal benefits that are not captured in conventional approaches: i.e. improved equity of access for isolated populations, access to specialist opinion, reduced travel requirements; unintended consequences may be captured by CBA as well |
| Whited 2010 | telemedicine | Review | availability of effectiveness (outcome) data; often only intermediate outcomes available; rapidly changing pricing structure of the technology; labour costs may be reduced in future by the adoption of computer algorithms/automated analyses; different equipment used may limit the generalizability of results; context dependency (distances of travel averted) | yes | n.m. | n.m. | n.m. | should be clearly stated, societal perspective recommended | rapidly changing prices of technology; technology may be used for other interventions as well |  | n.m. | reliable clinical outcome data is scarce so far | preferably clinical outcomes |
| Bergmo 2012 | telemedicine | Discussion | external validity (generalizability) due to heterogeneity and complexity of the field (diversity in terms of speciality, technology, applications, objectives and context); context variables have more impact on the results than the intervention itself; services can involve a complex mixture of technologies intermingled with different clinical an organizational routines and practices; specific challenges of pragmatic trials and decision analytic modelling | partially | pragmatic trials; decision analytic modelling | partially | complex intervention, many relevant context factors, multiple outcomes | has to be stated clearly in advance | cost should be assessed in pragmatic trials or different options should be analysed by decision analytic modelling |  | n.m. | decision modelling can be used to translate intermediate outcomes into final health outcomes | n.m. |
| Davalos; French; Burdick; Simmons 2009 | telemedicine | Discussion, Framework | research gaps: limited generalizability of studies (due to heterogeneity of telemedicine programs); disparate estimation methods; few completed CBAs; lack of RCTs; lack of long-term evaluation studies; absence of quality data and appropriate measures; small sample sizes (statistical limitations); CBAs are seldomly performed; difficulty of monetary valuation of specific aspects or outcomes of the interventions (i.e., faster diagnosis) | yes | CBA | yes (gold standard) | yes | societal perspective; disaggregated measures for client/provider/other stakeholders |  |  | covered by CBA | multidimensional outcomes for different stakeholders; outcomes such as patient’s health knowledge, ability for self-care, medication compliance, faster/accurate diagnosis and treatment, access to healthcare are difficult to value with currently available information (indirect effect of these outcomes on health improvement is not easy to identify) | detailed list of possible outcomes for clients, providers, other stakeholders that relate to the categories as medical effectiveness, employment, healthcare services and decreased travel is provided |
| Mair; Haycox; May; Williams 2000 | telemedicine | Recommendations | reliable evidence on effectiveness is scare so far; 'stepped' cost functions for telemedicine services (largely dependent on usage rates); marginal costs will differ between different patient groups: more evidence is necessary to estimate the cost functions adequality. | yes |  | yes |  | Analysis should include the perspective of service users and providers | see specific challenges; future changes in equipment and transmission costs should be included in sensitivity analyses |  | n.m. | evidence on benefits of telemedicine is inconclusive so far |  |
| McIntosh; Cairns 1997 | telemedicine | Framework | "(1) evaluation of constantly changing technology; (2) inadequate sample sizes; (3) limitations of the methodologies available for evaluation; (4) establishing an observable and empirical link between telemedicine and improved patient outcome; (5) inappropriateness of the conventional techniques of economic evaluation; (6) valuation of benefits, including non-health benefits, such as improvements in the process of care; (7) the short-term effects on infrastructure and organization may differ from the long-term effects. " (137) | no (CBA, CEA, CUA) | CCA (balance sheet - disaggregated costs and benefits); conjoint analysis for the valuation of non-health-benefits | yes | multidimensional outcomes | societal (differentiated by stakeholders) | high capital costs, low costs for each additional service; costs are difficult to estimate due to changes in costs of technology | equity/distribution of health care/access | yes | non-health outcomes are more important than direct health outcomes (but more difficult to value) | especially non-health outcomes: (1) improved quality of service, (2) transfer of skills, (3) speed of service, (4) education, (5) reassurance. |
| Bongiovanni-Delarozière; Le Goff-Pronost 2017 | telemedicine | Review, Framework | fast technological changes: sustainability of applications, the availability of outcome and cost data, the generalizability of results; shortcomings of classical economic evaluation methods in capturing multiple impacts of an intervention; difficulties of evaluating the start-up phase of a new medical practice; | partially | multidimensional assessment framework, including an impact matrix, combining quantitative and qualitative measures; CMA if health effects are equivalent | yes (with limitations) | yes | all stakeholders (including the industry) | inclusion of all stakeholders | accessibility, ethical and legal aspects | yes | multidimensional outcomes should be assessed by an impact matrix, that differentiates four categories of outcome indicators and four target groups; quantitative and qualitative approaches can be combined in this matrix | 4 categories: accessibility; professional practice/care organisation; care quality/safety; and costs  for 4 target groups: patients/family caregivers; health professionals; healthcare Institutions; government/health insurance/local authorities |
| Bergmo 2015 | eHealth (ICT in health care) | Tutorial | heterogeneity of the eHealth field: high diversity in technologies and applications, many different stakeholders; costs and effectiveness measures tend do be multifaceted, involve a wide range of effects on patients HC providers and society; generalizability of results is often limited (specificity of interventions) ; rapid development in the field limit generalizability; interventions are situated in complex systems that have the tendency to change, be self-organizing, be sensitive to initial conditions, and to behave in a nonlinear fashion | yes | adequate use of standard methodology is necessary, transparency in reporting the methodology is important to ensure comparability; costs and outcomes should be reported both at baseline and at every follow-up interval | no (naturalistic trials = gold standard) | yes | societal or health provider | finding appropriate cost weights and prices can be challenging (rapidly changing technology and prices); quantities of resources and cost weight should be reported separately to facilitate transferability in other contexts; the costs of supporting the health care providers in using the eHealth interventions should be included; |  |  | choosing one outcome measure for the CEA/CUA can miss important benefits; measurement and valuation of non-health consequences may be difficult (e.g., feelings of security, value of information, transfer of skill); | depends on the perspective, which and whose benefits to include is a normative issue and has to be decided in each specific evaluation |
| McNamee et al. 2016 | digital health interventions | Discussion | complex interventions in complex systems require more refined economic evaluation tools and methods; different possible aspects of complexity: intervention complexity (multiple interacting components, interventions change over time), outcomes complexity (spill-overs/externalities, feedback-loops); causal pathway complexity; effectiveness may depend on spread via social networks; selection of an appropriate modelling framework;  product reach and future costs of updating are highly unpredictable, and may be affected by regulatory changes | partially | type of method depends on research question and on the extent of interaction between intervention and system/setting or individuals; agent-based modelling approaches; decision theoretic approaches, social network analysis | not always appropriate |  | no recommendation (depends on intervention) | often high development costs and low maintenance costs; most DHIs require updates to remain 'the same'; many interventions evolve unpredictably over time; | n.m. | n.m. | some guidelines recommend surrogate measures for interventions that generate long-term effects - these may not be sufficient for DHIs that may adapt or change over time; relationship to 'final' measures is not well established | depend on the intervention; safety aspects (intentional and unintentional harms) have to be considered (i.e. outdated information in apps) |
| Crowe; Hailey; Carter 1992 | digital radiology systems | Discussion | few attempts so far do examine intangible effects (i.e. with respect to new types of staff and infrastructure), diverse range of stakeholders with different benefits/costs; studies on effectiveness of the systems have been inconclusive so far | partially | cost-benefit and social audit analysis techniques | yes | yes | multistakeholder-perspective (differentiated by the different parties involved) | intangible cost implications through new types of staff and infrastructure; (i.e. staff morale); costs to provide a secure operating environment are still unclear | equity, distributional consequences | yes (social audit analysis) | intangible benefits have to be considered; diverse benefits accrue to different stakeholders (thus a differentiated analysis in a cost benefit matrix is recommended) | Potential benefits are differentiated by stakeholder groups (diagnostician; referring physician; patient; hospital |
| Hailey 2005 | telemedicine | Discussion | good-quality evidence on clinical outcomes beyond the feasibility stage is scarce so far; availability of administrative and other data for conventional services is often limited; rapid evolution of the technology; limited generalizability of results due to setting specific factors | yes | CEA, CUA, social audit analysis, cost-consequence matrix | n.m. | n.m. | depends on research question | n.m. |  | n.m. |  | preferably clinical outcomes |
| NICE 2019 |  |  | diverse outcomes, non-health and outcomes that cannot be monetised | yes | BIA, CUA, CCA (depending on the maturity of the technology); if funded by NHS or Personal Social Services: CUA |  | yes | depending on decision-maker (payer or societal) |  | subgroup-analyses are recommended if clinical data indicates that effects differ by demographic factors | yes | diverse outcomes, non-health and outcomes that cannot be monetised | these include "patient benefits and related factors such as compliance, adverse events related to the DHT and ease of use for relevant staff group" (5) |
| NICE 2019 | digital health technologies | Evidence Standard Framework | not discussed | yes | BIA, CUA, CCA (depending on the maturity of the technology); if funded by NHS or Personal Social Services: CUA | yes, if applicable | n.m. | depending on decision-maker |  | subgroup-analyses are recommended if clinical data indicates that effects differ by demographic factors | CCA should be used if applicable | general recommendation (not specific for DHT) | "best quality evidence available" (27) |
| LeFevre et al. 2017 | m-health | discussion | Missing guidance on which analytical approaches are most appropriate based on the maturity of the mHealth solution (the paper tries to fill this gap); challenges depend on the different stages of development | yes | depending on stage and data availability; ECEA (extended CEA) and NBRF (net benefit regression framework) for subgroup analysis, equity and financial risk protection related research questions | not necessarily | n.m. | depending on the specific research question | Analysis should include economic and financial evaluation, including estimations on costs for sustainability/expansion and budget impact | equity, distributional consequences, financial risk protection | n.m. | not discussed | mainly health outcomes |
| Wildman; McMeekin; Grieve; Briggs 2016 | assisted living technologies (home/environment modifications & telemedicine) | methodology | inclusion of beyond-health and processual aspects in health economic methods, deciding on the appropriate study perspective and comparator, valuation of social care effects and QALYs, decide between divergent preferences of different stakeholder groups, integration of equity aspects | partially | combination of CUA and CBA, Q-methodology to elicit group views concerning relevant attributes | n.m. | yes | should be carefully considered | n.m. | equity | integrated by the proposed method | necessity of integrating beyond-health, health and processual aspects | social care effects, processual aspects and QALYs |
| Böhler 2018 |  |  | Main challenges are the rapid technological development, the potentially disruptive character of these technologies, their classification as complex interventions and the potential to generate non-health benefits is or benefits beyond the health care system; possibility of multiple, time-delayed, unpredictable, and difficult-to-assess (sometimes external) program effects; necessity of an early, iterative technology assessment from the perspective of different decision makers | partially/adapted | mainly CEA and CUA; CMA, CCA, MCDA should be considered depending on study question and perspective | yes | yes/often | differentiated by stakeholder; reference case: health system perspective | a variety of budgets of different stakeholders may be affected, multiple usage options; transferability of cost results during experimental studies to real-life applications is limited, disruptive character of the interventions | may be necessary | yes/often | non-health benefits; valuation of non-health benefits is often difficult | in general natural units and/or QALYs; if generic outcome measures are not adequate indication specific outcome measures should be considered. If there are considerable non-health benefits MCDA should be used |
| Hailey, David 2004 |  |  | data availability and analytic resources | yes | social audit analysis, cost-consequence matrix | n.m. | n.m. | depends on stage of development | costs per unit of service are hight at low caseloads, but will decline with volume of use; costs are expected to decline due to cheaper equipment costs in future | equity | yes, possible | n.m. | mainly health outcomes |
| Sisk; Sanders 1998 | telemedicine | Framework | multiple uses of the technology that are difficult to apportion to one service; the system may lead to expanded indications to use; dynamic process: development of technology (may decrease costs) and increased experience by user (may increase health benefits) | yes | standard methods; uncertainties should be addressed in sensitivity analyses | n.m. | n.m. | societal perspective is most relevant | see specific challenges | n.m. | n.m. | see specific challenges | health benefits or health outcomes (length/quality of life, physical, emotional, and social functioning; intermediate outcomes such as 'cases of disease diagnosed' may be acceptable |
| Jacobs; Barnett 2017 | general | Discussion | rapid technological changes; heterogeneity of telehealth interventions limits transferability and challenges model-based costing approaches; greater necessity to include patients’ perspectives (resources used by patients and caregivers) | yes (costing methods) | micro-costing for new technologies; iterative approach | n.m. | n.m. | inclusion of patient perspective is often relevant to accurately assess cost-effectiveness | disagreement on the inclusion of research and development costs; consideration of life-cycle of new technologies is necessary; costing studies should be updated throughout the different phases of a technology's life cycle (recommendation to use micro-costing methods, especially if no pricing information is available; decreasing prices have to be taken in account); | n.m. | n.m. | n.m. | n.m. |
